# Bruton's TK regulates myeloid cell recruitment during acute inflammation

**DOI:** 10.1111/bph.15778

**Published:** 2022-03-15

**Authors:** Gareth S. D. Purvis, Haidee Aranda‐Tavio, Keith M. Channon, David R. Greaves

**Affiliations:** ^1^ Sir William Dunn School of Pathology University of Oxford Oxford UK; ^2^ BHF Centre of Research Excellence University of Oxford Oxford UK; ^3^ Wellcome Centre for Human Genetics University of Oxford Oxford UK; ^4^ Department of Cardiovascular Medicine, Radcliffe Department of Medicine John Radcliffe Hospital Oxford UK

## Abstract

**Background and Purpose:**

Bruton's TK (BTK) is a non‐receptor kinase best known for its role in B lymphocyte development that is critical for proliferation and survival of leukaemic cells in B‐cell malignancies. However, BTK is expressed in myeloid cells, particularly neutrophils, monocytes and macrophages where its inhibition has been reported to cause anti‐inflammatory properties.

**Experimental Approach:**

We explored the role of BTK on migration of myeloid cells (neutrophils, monocytes and macrophages), in vitro using chemotaxis assays and in vivo using zymosan‐induced peritonitis as model systems.

**Key Results:**

Using the zymosan‐induced peritonitis model of sterile inflammation, we demonstrated that acute inhibition of BTK prior to zymosan challenge reduced phosphorylation of BTK in circulating neutrophils and monocytes. Moreover, pharmacological inhibition of BTK with ibrutinib specifically inhibited neutrophil and Ly6C^hi^ monocytes, but not Ly6C^lo^ monocyte recruitment to the peritoneum. X‐linked immunodeficient (XID) mice, which have a point mutation in the *Btk* gene, had reduced neutrophil and monocyte recruitment to the peritoneum following zymosan challenge. Pharmacological or genetic inhibition of BTK signalling substantially reduced human monocyte and murine macrophage chemotaxis, to a range of clinically relevant chemoattractants (C5a and CCL2). We also demonstrated that inhibition of BTK in tissue resident macrophages significantly decreases chemokine secretion by reducing NF‐κB activity and Akt signalling.

**Conclusion and Implications:**

Our work has identified a new role of BTK in regulating myeloid cell recruitment via two mechanisms, reducing monocyte/macrophages' ability to undergo chemotaxis and reducing chemokine secretion, via reduced NF‐κB and Akt activity in tissue resident macrophages.

## Graphical abstract

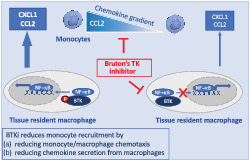

**Abbreviations**
ALL: acute lymphoblastic leukaemia
BMDMs: bone marrow‐derived macrophages
BTK: Bruton's TK
EMA: European Medicines Agency
FDA: Food and Drug Administration
PECs: peritoneal exudate cells
XID: X‐linked immunodeficient
XLA: X‐linked agammaglobulinemia
ZIP: zymosan‐induced peritonitis

**What is already known**

- BTK is involved in pro‐inflammatory neutrophil and macrophage signalling.

**What does this study add**

- BTK regulates myeloid cell recruitment by regulating monocytes/macrophage chemotaxis and macrophage chemokine production.

**What is the clinical significance**

- This gives insights into the off‐target effects of BTK inhibitors in myeloid cells on chemotaxis.

## 1 INTRODUCTION

Inflammatory cell recruitment is a key step in the initiation of the acute immune response. Macrophage activation at the site of tissue injury results in the secretion of chemokines; these have a non‐redundant role in leukocyte recruitment in preclinical models of inflammation. Activated macrophages are a major source of pro‐inflammatory cytokines and chemokines in many chronic inflammatory diseases including rheumatoid arthritis, diet‐induced diabetes or atherosclerosis (White et al., 2013). Chemokine‐mediated recruitment, retention and activation of leukocytes, are attractive areas for the development of novel anti‐inflammatory agents that could find application in a wide range of chronic inflammatory pathologies. We and others have demonstrated that genetic targeting of chemokine receptors shows promise in preclinical models of acute and chronic inflammation (McNeill et al., 2017). However, interventional studies in man using chemokine receptor antagonists have so far had limited therapeutic benefit (Noels et al., 2019; Schall & Proudfoot, 2011). This could be due to redundancy of chemokine receptor use in leukocytes to initiate monocyte/macrophage migration. Therefore, a therapeutic strategy that would affect both a myeloid cell's ability to undergo chemotaxis and simultaneously reduce chemokine secretion could limit leukocyte recruitment by two independent but complementary mechanisms.

Bruton's TK (BTK) is a non‐receptor bound intracellular signalling molecule best known for its signalling in B‐cell development and malignancy. However, in recent years, an alternative role for BTK is emerging in innate immune cells. BTK has been shown to be expressed at relatively high levels in myeloid cells, specifically monocytes, neutrophils and macrophages (Mangla et al., 2004). BTK is activated in monocytes and macrophages in numerous acute inflammatory conditions, including polymicrobial sepsis and cerebral ischaemia (Ito et al., 2015; O'Riordan et al., 2019) but also in chronic inflammatory conditions such as obesity‐induced diabetes, rheumatoid arthritis and lupus (Hartkamp et al., 2015; Honigberg et al., 2010; Purvis et al., 2020). We and others have reported inhibition of BTK reduces NF‐κB activity in murine and human macrophages following LPS stimulation and after *Aspergillus fumigatus* infection (Bercusson et al., 2018; Purvis et al., 2020). Work by Ní Gabhann et al. (2014) reported a role for BTK in macrophage M1 polarisation, which was dependent on STAT1 and p65 phosphorylation, while O'Riordan et al. (2020) demonstrated that X‐linked immunodeficient (XID) mice subjected to polymicrobial sepsis had reduced M1 macrophages compared with wild‐type (WT) controls due to reduced NF‐κB and NLRP3 inflammasome activation. Following NF‐κB activation, components of the NLPR3 inflammasome pathway are also up‐regulated (Liu, Zhang, et al., 2017). BTK has been shown to be a regulator of ACS spec formation in murine and human macrophages (Ito et al., 2015), and the proteolytic release of IL‐1β ad IL‐18, via caspase 1 activity. In addition, ibrutinib blocks the secretion of IL‐1β in cells derived from patients with Muckle–Wells syndrome (Liu, Pichulik, et al., 2017). Indeed, during the COVID‐19 pandemic, BTK has been shown to be activated in monocytes and intervention with the BTK inhibitor acalabrutinib has been reported to reduce systemic inflammation in patients with severe COVID‐19 (Roschewski et al., 2020). Nicolson, Welsh, et al. (2020) provided evidence that BTK inhibitors could be used to reduced thromboinflammation in COVID‐19 patients (Siess et al., 2020).

Pharmacological inhibition of BTK reduced macrophage accumulation in inflamed tissues in a preclinical model of type II diabetes (Purvis et al., 2020) and a model of cerebral ischaemia (Ito et al., 2015). To confirm and extend these studies, we tested the hypothesis that BTK can directly regulate myeloid cell recruitment to sites of inflammation. To do this, we used a combination of in vivo and in vitro cell recruitment assays using a range of European Medicines Agency (EMA) or US Food and Drug Administration (FDA) approved BTK inhibitors and tool compounds in combination with BTK‐deficient XID mice. This allowed us to fully explore the magnitude and kinetics of myeloid cell recruitment in acute inflammation. We demonstrate that inhibition of BTK activity (phosphorylation) reduces neutrophil and Ly6C^hi^ monocyte recruitment via two independent but complementary mechanisms: (a) reducing monocyte chemotaxis and (b) reducing chemokine production from tissue resident macrophages.

## 2 METHODS

### 2.1 Animals

Animal studies are reported in compliance with the ARRIVE guidelines (Percie du Sert et al., 2020) and with the recommendations made by the *British Journal of Pharmacology* (Lilley et al., 2020). All animal studies were conducted with ethical approval from the Dunn School of Pathology Local Ethical Review Committee and in accordance with the UK Home Office regulations (Guidance on the Operation of Animals, Scientific Procedures Act, 1986). Male (8–12 weeks) C57BL/6J (RRID:IMSR_JAX:000664), CBA/CaCrl (RRID:MGI:5659142; WT strain for XID mice) mice were obtained from Charles River Laboratories (Oxfordshire, UK). XID mice (CBA/CaHN‐*Btk* ^xid^/J; RRID:IMSR_JAX:00101) (Lindsley et al., 2007) are an inbred strain on the CBA background purchased from The Jackson Laboratory. They have a point mutation rendering the kinase domain of BTK inactive. Specifically, there is a C to T substitution at coding nucleotide 82, which alters the amino acid sequence, substituting an arginine for cysteine. The substitution is in a conserved PH domain and blocks the activation of the kinase (Rawlings et al., 1993) preventing BTK phosphorylation at Tyr^223^, which is a key activating site. Importantly, ibrutinib binds irreversibly to Cys^481^, also in the active site of the kinase domain, and inhibits auto‐phosphorylation of Tyr^223^, thus blocking BTK activity. All mice were then housed in the same unit under conventional housing conditions at 25 ± 2°C and had access to food and water ad libitum.

### 2.2 Zymosan‐induced peritonitis and flow cytometry

C57BL/6J, CBA/CaHN‐*Btk* ^xid^/J (XID) or CBA/CaCrl (background strain for XID mice, and thus used as the WT for XID mice) or mice were administered 100‐μg zymosan A (Sigma‐Aldrich) in PBS or vehicle (500 μl; PBS) intraperitoneally. After 2, 4, 16 or 48 h, mice were killed by increasing concentrations of CO_2_ and exsanguination, and peritoneal exudates collected by lavage with 7 ml of ice‐cold sterile PBS with 5‐mM EDTA. Total cell counts and cellular composition of peritoneal exudate were determined as previously described (Cash et al., 2009). Antibodies that were used to identify neutrophils (CD11b^+^LyGC^+^Ly6G^+^), monocytes (CD11b^+^LyGC^+^Ly6G^−^), pro‐inflammatory monocytes (CD11b^+^Ly6G^−^CD115^+^Ly6C^hi^), patrolling monocytes (CD11b^+^Ly6G^−^CD115^+^Ly6C^lo^) and B‐cells (CD11b^−^BV220^+^) were purchased from BioLegend (San Diego, CA, USA). Full gating strategy is provided in Figure S1.

Cell surface expression of chemokine receptors on monocytes (CCR2: AB_2810414, BioLegend, Cat. No. 150627), neutrophils (CXCR4: AB_2734225, BioLegend, Cat. No. 153805) and bone marrow‐derived macrophage (BMDM) (C5aR: AB_2067285, BioLegend, Cat. No. 135805) antibodies were all purchased from BioLegend. Additionally, intracellular staining for phosphorylated BTK protein was performed. Briefly, cell surface expression on relevant cell types was carried out on ice, neutrophils (CD11b^+^CD115^−^Ly6G^+^), Ly6C^hi^ monocytes (CD11b^+^Ly6G^−^CD115^+^Ly6C^hi^) and Ly6C^lo^ monocytes (CD11b^+^Ly6G^−^CD115^+^Ly6C^lo^) and resident peritoneal macrophages ex vivo. Cells were then spun and washed in Fix/Perm (eBioSciences) for 30 min at room temperature (RT). Cells were then washed in Permeabilisation Buffer (eBioSciences), before Tyr^223^ on BTK antibody (AB_2721620: BioLegend, Cat. No. 601703) was added for 30 min, followed by washing in Permeabilisation Buffer and fixing in 4% methanol‐free paraformaldehyde. Data were acquired using a BD LSRFortessa cytometer, and data analysed using FlowJo (Version 10). Full gating strategies can be found in Figure S1.

Dosing regimen was determined from assessment of previously published doses of ibrutinib known to inhibit BTK in vivo (O'Riordan et al., 2019); pre‐dosing strategy was used as a proof of principle methodology for drug efficacy. Delivery route (oral gavage) has been previously demonstrated.

#### 2.2.1 Ibrutinib pretreatment dose response in 16‐h zymosan‐induced peritonitis (ZIP)

Male C57BL/6J mice (8 weeks old) were pretreated with ibrutinib (0.01, 0.03, 0.1, 0.3, 1, 3 or 10 mg·kg^−1^; p.o.) or vehicle (5% DMSO, 30% cyclodextrin) 1 h prior to zymosan challenge (100 μg; i.p.). Peritoneal exudate cells (PECs) were harvested in ice‐cold cell PEC harvest buffer (PBS, 5‐mM EDTA) 16 h after zymosan challenge.

#### 2.2.2 Ibrutinib pretreatment 48‐h ZIP time course

Male C57BL/6J mice (10–12 weeks old) were pretreated with ibrutinib (1 mg·kg^−1^; p.o.) or vehicle (5% DMSO, 30% cyclodextrin) 1 h prior to zymosan challenge (100 μg; i.p.). PECs were harvested in ice‐cold PEC harvest buffer (PBS, 5‐mM EDTA) 2, 4, 16 and 48 h after zymosan challenge.

#### 2.2.3 BTK inhibitors in 16‐h ZIP model

Male C57BL/6J mice (10–12 weeks old) were pretreated with ONO‐4059, CNX‐774, olmutinib, LFM‐A13 and acalabrutinib (1 mg·kg^−1^; p.o.) or vehicle (5% DMSO, 30% cyclodextrin) 1 h prior to zymosan challenge (100 μg; i.p.). Compounds were all purchased from SelleckChem, as selected because they had varying properties compared with ibrutinib, that is, more selective (acalabrutinib), not marketed as a BTK inhibitor but targets BTK (olmutinib), more potent than ibrutinib (ONO‐4059), structurally different to ibrutinib (CNX‐774). All compounds are also reported to be bioactive following oral gavage. Blood was collected by direct cardiac puncture post mortem and PECs were harvested in ice‐cold cell PEC harvest buffer (PBS, 5‐mM EDTA) 16 h after zymosan challenge.

#### 2.2.4 WT versus XID mice in 16‐h ZIP model

Male CBA/CaCrl (WT) or XID mice (8–10 weeks old) were given zymosan challenge (100 μg; i.p.). Blood was collected by direct cardiac puncture post mortem; PECs were harvested in ice‐cold PEC harvest buffer (PBS, 5‐mM EDTA) and spleen dissected 16 h after zymosan challenge.

### 2.3 Murine BMDMs

BMDMs were generated as previously described (Recio et al., 2018). Briefly, fresh bone marrow cells from tibiae and femurs of male C57BL/6J or XID mice aged 8–10 weeks were cultured in DMEM containing 4.5 g·L^−1^ glucose, 2‐mM l‐glutamine, 50 units·ml^−1^ penicillin and 50 μg·ml^−1^ streptomycin, 10% heat‐inactivated FBS, 10% L929 cell‐conditioned media (as a source of macrophage colony‐stimulating factor) and for 7 days. Bone marrow cells were seeded into 8 ml of medium in 90‐mm non‐tissue culture treated Petri dishes (ThermoFisher Scientific, Sterilin, UK). On Day 5, an additional 5 ml of medium was added. Gentle scraping was used to lift cells. BMDMs were then counted and suspended in FBS free media at the desired cell concentration.

### 2.4 Murine resident peritoneal macrophages

PECs were harvested in ice‐cold PEC harvest buffer (PBS, 5‐mM EDTA); cells were then pelleted and resuspended in DMEM containing 4.5 g·L^−1^ glucose, 2‐mM l‐glutamine, 50 units·ml^−1^ penicillin and 50 μg·ml^−1^ streptomycin, 10% heat‐inactivated FBS and allowed to attach to tissue culture‐treated plastic for 1 h. Unattached cells were washed off and cells stimulated as per experimental protocol.

### 2.5 Human monocyte isolation

Human blood was obtained from healthy donors with informed consent and ethical approval in the form of leukocyte cones purchased from the NHS Blood and Transplant service. Leukocyte cones contain waste leukocytes isolated from individuals donating platelets via apheresis and consist of a small volume (~10 ml) of packed leukocytes with few red blood cells or platelets. For monocyte isolation, blood was diluted with 1:2 with PBS followed by separation using a Histopaque gradient and centrifugation as previously described (Purvis et al., 2020). Following human peripheral blood mononuclear cell (PBMC) isolation and washing, approximately 1 × 10^8^ PBMCs were labelled and negatively selected using the pan human monocytes isolation kit and MACS separation (Miltenyi Biotec, Bisley, Surrey, UK), typically yielding around 5 × 10^6^ monocytes defined as CD11b^+^CD114^+^CD16^−^. Monocytes were resuspended at 4 × 10^6^ cells·ml^−1^ in chemotaxis buffer (RPMI 1640, 25‐mM HEPES, 0.5% [w/v] BSA) and left on ice until required.

### 2.6 ACEA xCELLigence real‐time cell migration

Experiments were carried out with CIM‐16 plates and an xCELLigence RTCA‐DP instrument (ACEA, San Diego, USA) as previously described (Iqbal et al., 2013). Chemoattractants (complement C5a and CCL2, both purchased from Peprotech, UK) were made to desired concentrations (10 nM) in chemotaxis buffer (RPMI 1640/25‐mM HEPES/0.5% [w/v] BSA) and loaded into the lower wells of the CIM‐16 plate. Upper wells were filled with chemotaxis buffer and plates equilibrated for 30 min at RT. BMDM or human monocytes were resuspended in chemotaxis buffer and incubated with ibrutinib (1–30 μM) or vehicle (0.3% DMSO) for 60 min at 37°C, 5% CO_2_. Cell suspensions (BMDM; 2 × 10^6^/ml; hMonocytes; 4 × 10^6^/ml) were placed into the wells of the upper chamber, and the assay performed with recording of cellular impedance every 15 s for up to 8 h.

### 2.7 Macrophage NF‐κB reporter cell line (RAW Blue)

A commercial macrophage reporter cell line (RAW Blue cells; InvivoGen, San Diego, CA, USA; RRID:CVCL_X594) was used: Briefly, cells were derived from murine RAW 264.7 macrophages with chromosomal integration of a secreted embryonic alkaline phosphatase (SEAP) reporter construct, induced by NF‐κB and activator protein 1 (AP‐1) transcriptional activation. Cells were grown to 80% confluence in DMEM containing 4.5 g·L^−1^ glucose, heat‐inactivated 10% FBS, 2‐mM l‐glutamine and 200 μg·ml^−1^ Zeocin antibiotic (InvivoGen) at 37°C in 5% CO_2_. To minimise experimental variability, only cells with fewer than five passages were used. Cells were plated at 0.95 × 10^5^ per well of a 96‐well plate. After experimental treatments, cell supernatants were added to 180 μl of QuantiBlue substrate (InvivoGen) and incubated at 37°C for 60 min, and the plate read at an OD 655 nm (OD_655_) on a microplate spectrophotometer (PherastarFSX, BMG Lab, UK).

### 2.8 MTT assay

RAW Blue cell line viability was assessed by the mitochondrial metabolisation of the tetrazolium salt 3‐(4,5‐methyltiazol‐2yl‐)‐2,5diphenyl‐tetrazolium bromide (MTT) (AppliChem, Germany) colorimetric assay. Cells were maintained and plated as explained previously. The same treatment used for QuantiBlue assay was performed. Then MTT (0.5 mg·ml^−1^) was added, and cells were incubated at 37°C for 2 h. Cell media were removed and formazan crystals were dissolved in DMSO. OD was measured at 570 nm (OD_570_) on a microplate spectrophotometer (PherastarFSX, BMG Lab).

### 2.9 ELISA

Measurement of the protein levels of CXCL1 and CCL2 from peritoneal lavage fluids or secreted into cell supernatants from 1.5 × 10^6^ resident peritoneal macrophages was performed by ELISA (R&D Systems) according to the manufacturer's instructions.

### 2.10 Western blotting

The immuno‐related procedures used comply with the recommendations made by the *British Journal of Pharmacology* (Alexander, Christopoulos, et al., 2021). Cells were lysed by adding RIPA buffer (Sigma‐Aldrich) supplemented with protease and phosphatase inhibitors (Sigma, UK) followed by manual disruption (Purvis et al., 2019). Protein concentration was determined by using a BCA protein assay kit (Thermo Fisher Scientific). Total cell protein (20 μg) was added to 4× Laemmli buffer (250‐mM Tris–HCl, pH 6.8, 8% SDS, 40% glycerol, 0.004% bromophenol blue, 20% β‐mercaptoethanol) and heated at 72°C for 10 min. Samples were then resolved on SDS‐PAGE gels and transferred onto Hybond ECL nitrocellulose membranes (GE Healthcare, Buckinghamshire, UK). Membranes were blocked with 5% milk in Tris‐buffered saline with Tween (TBS‐T) (Tris‐buffered saline, 0.1% Tween‐20) for 1 h at RT and then incubated with the primary antibody: rabbit anti‐phospho‐BTK (RRID:AB_2800099, Cell Signaling Technology, Cat# 87141), rabbit anti‐total BTK (RRID:AB_10950506, Cell Signaling Technology, Cat# 8547), rabbit anti‐phospho‐Akt (RRID:AB_331159, Cell Signaling Technology, Cat#4051) and rabbit anti‐total Akt (RRID:AB_329827, Cell Signaling Technology, Cat#9272) diluted 1:1000 in 5% BSA/TBS‐T overnight at 4°C. Next, membranes were incubated with an HRP‐conjugated anti‐rabbit secondary antibody (RRID:AB_2099233, Cell Signaling Technology Cat# 7074, or RRID:AB_330924, Cell Signaling Technology, Cat# 7076) for 1 h at RT. Protein bands were visualised by incubating the membranes for 5 min with Amersham ECL prime and subsequent exposure to X‐ray film over a range of exposure times. Western blotting of stripped membranes with an anti‐β‐actin antibody (Cell Signaling) was used as a loading control. Densitometry was preformed using the Licor Studio Lite software.

### 2.11 Statistical analysis

The data and statistical analysis comply with the recommendations of the *British Journal of Pharmacology* on experimental design and analysis in pharmacology (Curtis et al., 2018). All experiments were designed, where possible, to generate groups of equal size. Power calculations were used to estimate the group size based on an expected effect size of 30%. Where possible, blinding and randomisation protocols were used. All data in the text and figures are presented as mean ± SEM of *n* observations, where *n* represents the number of animals studied (in vivo) or independent values, not technical replicates (in vitro). Exclusion criteria were pre‐determined before the beginning of experiment, if there was blood in the peritoneal lavage at harvest an individual *n* would be excluded, or at analysis stage outliners were defined as > ±2 SD from the mean. For western blot data, all data are represented as fold change compared with mean control. All statistical analysis was performed using GraphPad Prism 7 for Mac (GraphPad Software, San Diego, CA, USA; RRID:SCR_002798). Statistical analysis was only undertaken for studies where each group size was at least *n* = 5. For western blot analysis, representative data are shown where group size is *n* = 3. When the mean of two experimental groups was compared, a two‐tailed Student's *t*‐test was performed. Normally distributed data without repeated measurements were assessed by a one‐way ANOVA followed by Bonferroni correction if the *F* value reached significance at *P* < 0.05 level, and there was no significant variance inhomogeneity. In all cases, a *P* < 0.05 was deemed significant.

### 2.12 Materials

All chemicals and reagents were supplied from Merck Life Science UK Limited, The Old Brickyard, New Rd, Gillingham, Dorset SP8 4XT, UK, unless otherwise stated. All antibodies for flow cytometry were supplied by BioLegend, 33 Greenwood Place, London NW5 1LB, UK. Ibrutinib and acalabrutinib were supplied by Cambridge Biosciences, Munro House, Trafalgar Way, Bar Hill, Cambridge CB23 8SQ, UK. ONO‐4059, CNX‐774, Olmutinib and LFM‐A13 were supplied by Selleck Chemicals GmbH, Karl‐Schmid‐Str. 14, 81829 Munich, Germany.

### 2.13 Nomenclature of targets and ligands

Key protein targets and ligands in this article are hyperlinked to corresponding entries in the IUPHAR/BPS Guide to PHARMACOLOGY http://www.guidetopharmacology.org and are permanently archived in the Concise Guide to PHARMACOLOGY 2021/22 (Alexander, Christopoulos, et al., 2021; Alexander, Fabbro, et al., 2021a, 2021b)

## 3 RESULTS

### 3.1 Pretreatment with ibrutinib prior to zymosan challenge reduces myeloid cell recruitment to the peritoneum

**FIGURE 1:**
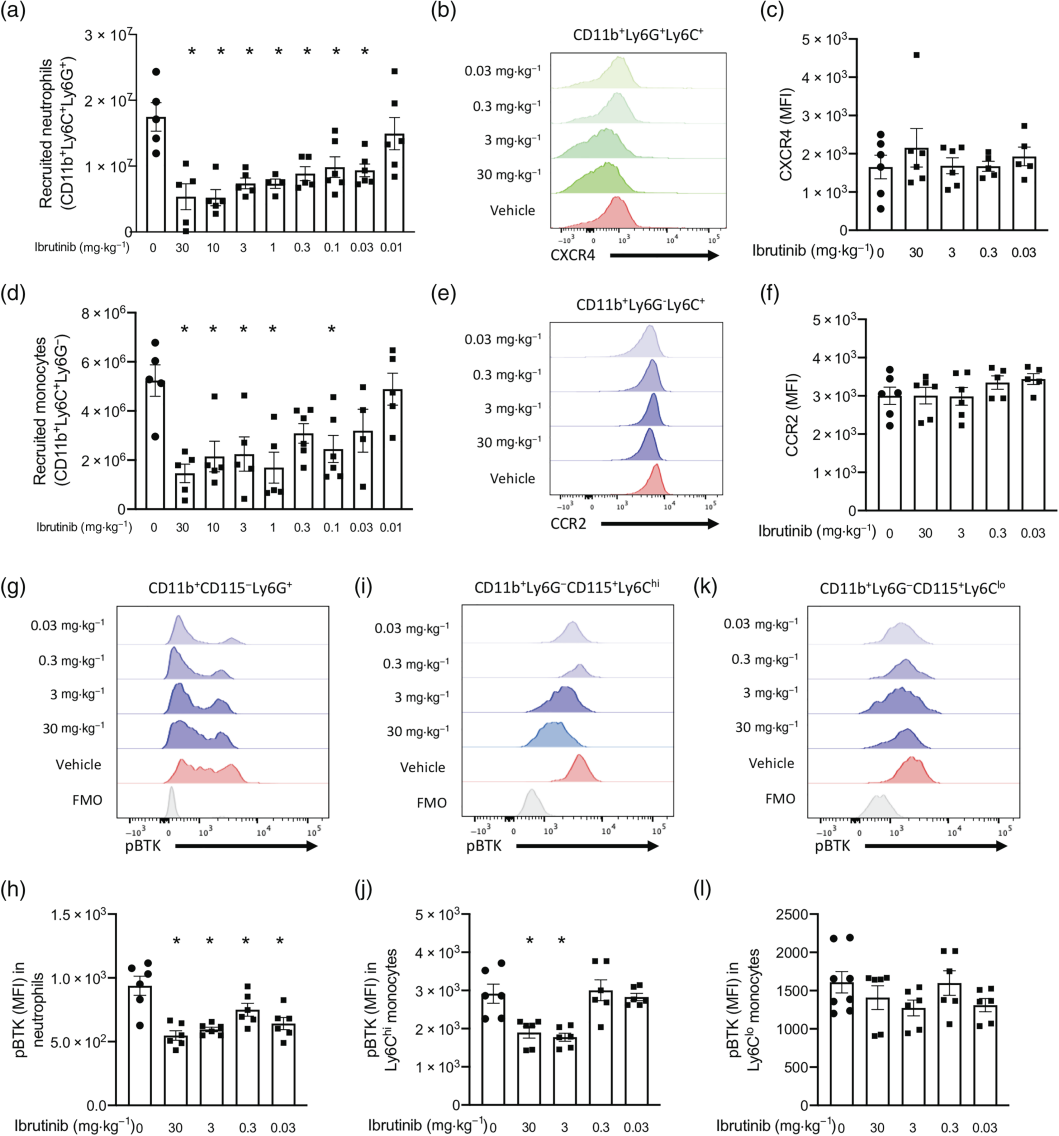
Inhibition of BTK with ibrutinib 1 h prior to zymosan challenge reduces myeloid cell recruitment to the peritoneum. C57BL/6J mice were pretreated with increasing doses of ibrutinib (0.01–30 mg·kg^−1^; p.o.) 1 h prior to zymosan challenge (100 μg; i.p.) and peritoneal exudate cells harvested after 16 h. (a) Number of recruited neutrophils (CD11b^+^Ly6C^+^Ly6G^+^), (b) representative histograph of mean fluorescent intensity (MFI) of CXCR4 on recruited neutrophils (CD11b^+^Ly6C^+^Ly6G^+^) and (c) quantification of neutrophil CXCR4. (d) Number of recruited monocytes (CD11b^+^Ly6C^+^Ly6G^−^), (e) representative histograph of mean fluorescent intensity (MFI) of CCR2 on recruited monocytes (CD11b^+^Ly6C^+^Ly6G^−^) and (f) quantification of monocyte CCR2. (g) Representative histograph of mean fluorescent intensity (MFI) tyr^223^ phosphorylation of BTK (pBTK) on blood neutrophils (CD11b^+^CD115^−^Ly6G^+^) and (h) quantified. (i) Representative histograph of mean fluorescent intensity (MFI) tyr^223^ phosphorylation of BTK on blood Ly6C^hi^ monocytes (CD11b^+^Ly6G^−^CD115^+^Ly6C^hi^) and (j) quantified. (k) Representative histograph of mean fluorescent intensity (MFI) tyr^223^ phosphorylation of BTK on blood Ly6C^lo^ monocytes (CD11b^+^Ly6G^−^CD115^+^Ly6C^lo^) and (l) quantified. Data shown are means ± SEM of *n* = 5 or 6 biological replicates. *P < 0.05 versus vehicle only; one‐way ANOVA was performed with Bonferroni post hoc test

ZIP (using 100 μg of zymosan) is a model of acute inflammation characterised by the recruitment of myeloid cells to the peritoneum following injection of zymosan that is largely resolved after 48–96 h (Regan‐Komito et al., 2017). To test if pharmacological inhibition of BTK with the FDA/EMA approved BTK inhibitor ibrutinib would reduce myeloid cell recruitment during acute inflammation, C57BL/6J mice were pretreated with ibrutinib (0.01–30 mg·kg^−1^; p.o.) 1 h prior to zymosan (100 μg; i.p.). Total PECs were harvested 16 h later and analysed using multicolour flow cytometry. Total cellularity within the peritoneum increased significantly within 16 h of zymosan challenge (data not shown), and this was mainly composed of neutrophils (CD11b^+^Ly6C^+^Ly6G^+^) (Figure 1a) and monocytes (CD11b^+^Ly6C^+^Ly6G^−^) (Figure 1d). As there is a resident population of B‐cells in the peritoneum, we also assessed peritoneal B‐cell number (Figure S2A). Ibrutinib treatment decreased total cellularity; specifically, there was a dose‐dependent decrease in neutrophil recruitment to the peritoneum at 16 h (Figure 1a). We next investigated if ibrutinib pretreatment prior to zymosan challenge affected chemokine receptor expression levels on the cells surface of recruited neutrophils. Importantly, ibrutinib pretreatment did not alter expression levels of CXCR4 on recruited CD11b^+^Ly6C^+^Ly6G^+^ neutrophils (Figure 1b/c). Pretreatment with ibrutinib prior to zymosan challenge elicited a dose‐dependent decrease in monocytes recruitment (Figure 1d) into the peritoneum at 16 h. Ibrutinib pretreatment specifically decreased Ly6C^hi^ monocyte recruitment in a dose‐dependent manner (Figure S2B), while there was no significant decrease in the recruitment of Ly6C^lo^ monocytes. Ibrutinib pretreatment prior to zymosan challenge did not alter expression levels of CCR2 on recruited CD11b^+^Ly6C^+^Ly6G^−^ monocytes (Figure 1e/f). Additionally, ibrutinib pretreatment prior to zymosan challenge did not alter B‐cells numbers within the peritoneum (Figure S1A). These results clearly demonstrate a direct role for BTK in myeloid cell recruitment in vivo, which is independent of chemokine receptor expression.

We next wanted to assess the level of BTK activation in ibrutinib‐treated mice in cells prior to recruitment to the peritoneum. To do this, we isolated peripheral blood cells and performed intracellular staining for the phosphorylation of Tyr^223^ on BTK in CD11b^+^CD115^−^Ly6G^+^ neutrophils, CD11b^+^Ly6G^−^CD115^+^Ly6C^hi^ monocytes and CD11b^+^Ly6G^−^CD115^+^Ly6C^lo^ monocytes. Ibrutinib pretreatment prior to zymosan challenge reduced phosphorylation of Tyr^223^ on BTK in CD11b^+^CD115^−^Ly6G^+^ neutrophils in the blood at 16 h (Figure 1g/h). Similarly, there was also a reduction in phosphorylation of Tyr^223^ on BTK in CD11b^+^Ly6G^−^CD115^+^Ly6C^hi^ monocytes (Figure 1i/j). However, when phosphorylation of Tyr^223^ on BTK in CD11b^+^Ly6G^−^CD115^+^Ly6C^lo^ monocytes was assessed, no reduction was seen in ibrutinib‐treated mice (Figure 1k/l). These results reflect those seen in the recruited monocytes populations, whereby ibrutinib pretreatment significantly reduced Ly6C^hi^ monocyte recruitment but did not have an effect on Ly6C^lo^ monocyte recruitment to the peritoneum following zymosan challenge.

Collectively, these results point to intracellular BTK signalling being important in neutrophil and Ly6C^hi^ monocyte recruitment from the blood to the peritoneum, and these effects are independent of chemokine receptor expression.

### 3.2 Ibrutinib treatment 1 h prior to zymosan challenge reduces myeloid cell recruitment over a 48‐h time course

**FIGURE 2:**
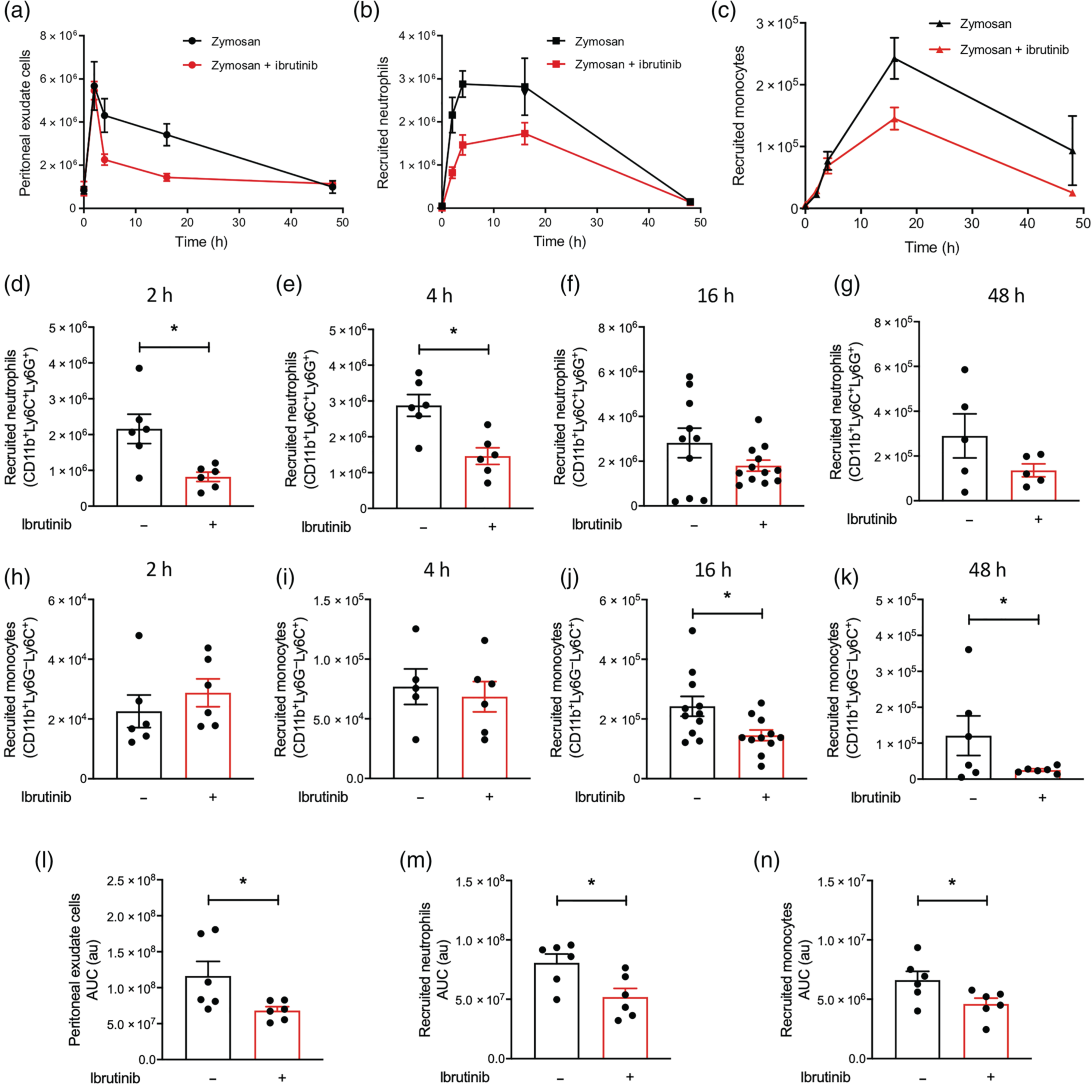
Time course of peritoneal myeloid cell recruitment in mice treated with ibrutinib. C57BL/6J mice were pretreated with ibrutinib (10 mg·kg^−1^; p.o.) or vehicle 1 h prior to zymosan challenge (100 μg; i.p.) and peritoneal exudate cells harvested after 2, 4, 16 and 48 h after zymosan challenge. (a) Total cell count in peritoneal exudate and quantified in (l), (b) number of recruited neutrophils (CD11b^+^Ly6C^+^Ly6G^+^) and quantified in (m) and (c) recruited monocytes (CD11b^+^Ly6C^+^Ly6G^−^) and quantified in (n). Recruited neutrophils (CD11b^+^Ly6C^+^Ly6G^+^) quantified at (d) 2, (e) 4, (f) 16 and (g) 48 h. Recruited monocytes (CD11b^+^Ly6C^+^Ly6G^−^) quantified at (h) 2, (i) 4, (j) 16 and (k) 48 h. Data shown are means ± SEM, *n* = 5–12 mice per group. *P < 0.05 versus vehicle only; a Student's *t*‐test was performed when two groups are compared

Having shown that ibrutinib treatment reduced both neutrophil and monocyte recruitment at 16 h, we wanted to understand the full kinetics of myeloid cell recruitment over a 48‐h time period. Mice were given ibrutinib (1 mg·kg^−1^; p.o.) 1 h prior to zymosan challenge and total PECs harvested at 2, 4, 16 and 48 h. Following zymosan challenge, there is rapid recruitment of cells into the peritoneum within the first 4 h (Figure 2a), of which the majority are Ly6C^+^Ly6G^+^ neutrophils (Figure 2b); this is followed by the peak infiltration of Ly6C^+^Ly6G^−^ monocytes at 16 h (Figure 2c). Ibrutinib treatment 1 h prior to zymosan challenge significantly reduced peak neutrophil recruitment at 2 and 4 h (Figure 2d/e). The initial peritoneal monocyte recruitment from the blood monocytes (2 and 4 h) was unaltered in ibrutinib‐treated mice (Figure 2h,i), but BTK inhibition significantly reduced the peak monocyte recruitment at 16 h and was maintained up to 48 h (Figure 2j,k). Overall, the magnitude of cellular recruitment is significantly reduced in mice given a single ibrutinib treatment prior to zymosan challenge compared with mice given vehicle prior to zymosan challenge over the entire 48‐h time frame studied (Figure 2l,m).

### 3.3 Multiple BTK inhibitors reduce myeloid cell recruitment to the peritoneum and reduce recruitment to the blood from the spleen

**FIGURE 3:**
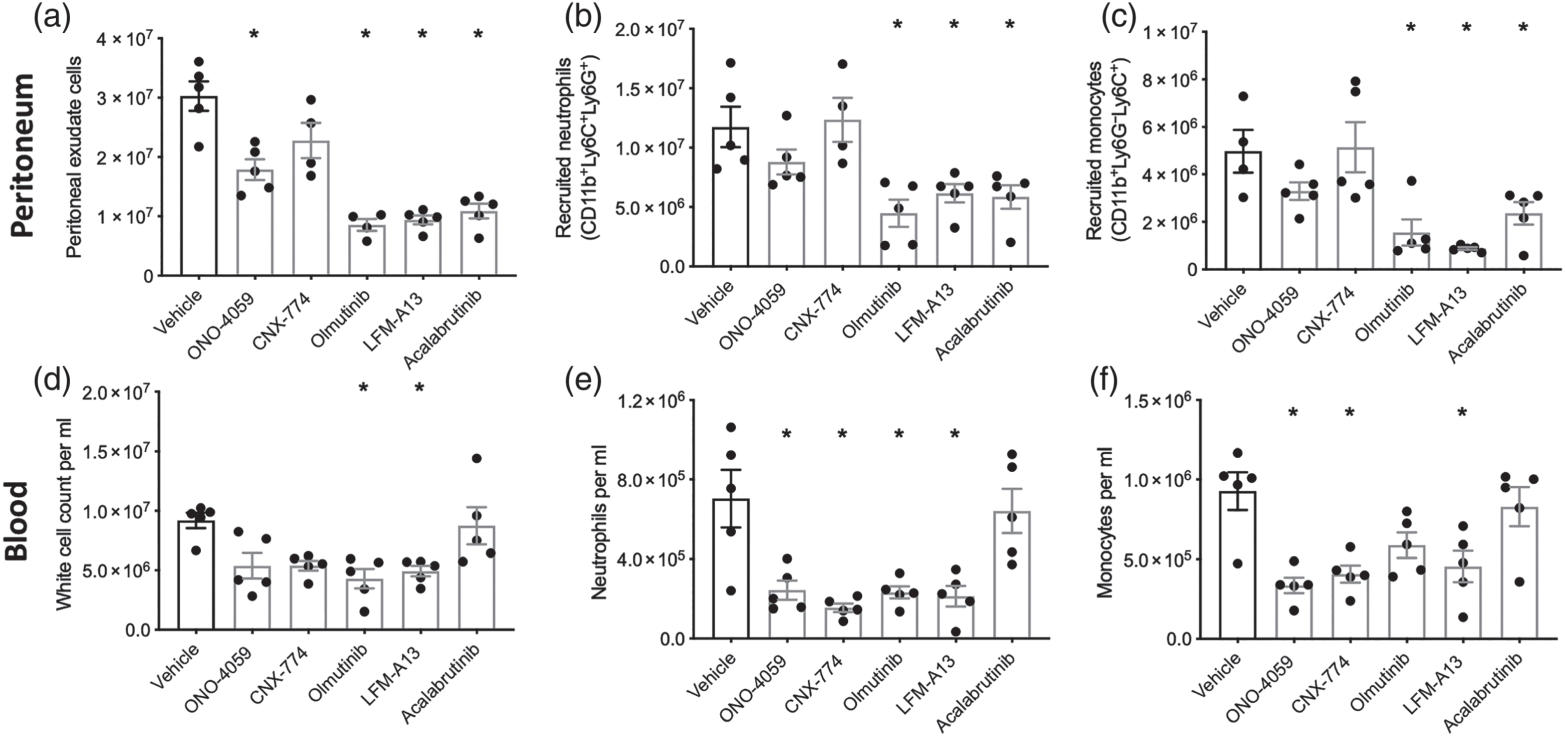
Inhibition of BTK reduces myeloid cell recruitment to the peritoneum and reduces recruitment to the blood. (a–c) C57BL/6J mice were pretreated with a range of BTK inhibitors (1 mg·kg^−1^; p.o.) 1 h prior to zymosan challenge (100 μg; i.p.) and peritoneal exudate cells were harvested at 16 h. (a) Total peritoneal exudate cells were quantified, (b) recruited neutrophils (CD11b^+^Ly6C^+^Ly6G^+^) and (c) recruited monocytes (CD11b^+^Ly6C^+^Ly6G^−^). (d–f) Blood was harvested (d) total cellular count, (e) neutrophils (CD11b^+^Ly6C^+^Ly6G^+^) and (f) monocytes (CD11b^+^Ly6C^+^Ly6G^−^) counts assessed. Data shown are means ± SEM of *n* = 5 mice per group. *P < 0.05 versus vehicle only; one‐way ANOVA was performed with Bonferroni post hoc test

Having shown that ibrutinib, the first BTK inhibitor to gain FDA approval, inhibits neutrophil and monocyte recruitment to the peritoneum, we wanted to test a range of both more selective (e.g., acalabrutinib) and less potent or more/less selective commercially available BTK inhibitors. All BTK inhibitors tested reduced cellularity in the peritoneum 16 h after zymosan challenge (Figure 3a). This was largely due to reductions in neutrophil accumulation (Figure 3b), while there was a similar trend in reducing monocyte accumulation (Figure 3c). We also measured myeloid cell numbers in the blood. Following zymosan challenge, neutrophils and monocytes are mobilised to the blood (Figure 3d). Overwhelmingly, mice treated with a BTK inhibitor prior to zymosan challenge had fewer neutrophils (Figure 3e) and monocytes (Figure 3f) in the blood at 16 h, the exception being acalabrutinib pretreatment that did not significantly alter neutrophil or monocyte mobilisation to the blood. These results collectively point to a central role of BTK in the regulation of neutrophil and monocyte recruitment in vivo.

### 3.4 XID mice have reduced myeloid cell recruitment to the peritoneum following zymosan challenge

**FIGURE 4:**
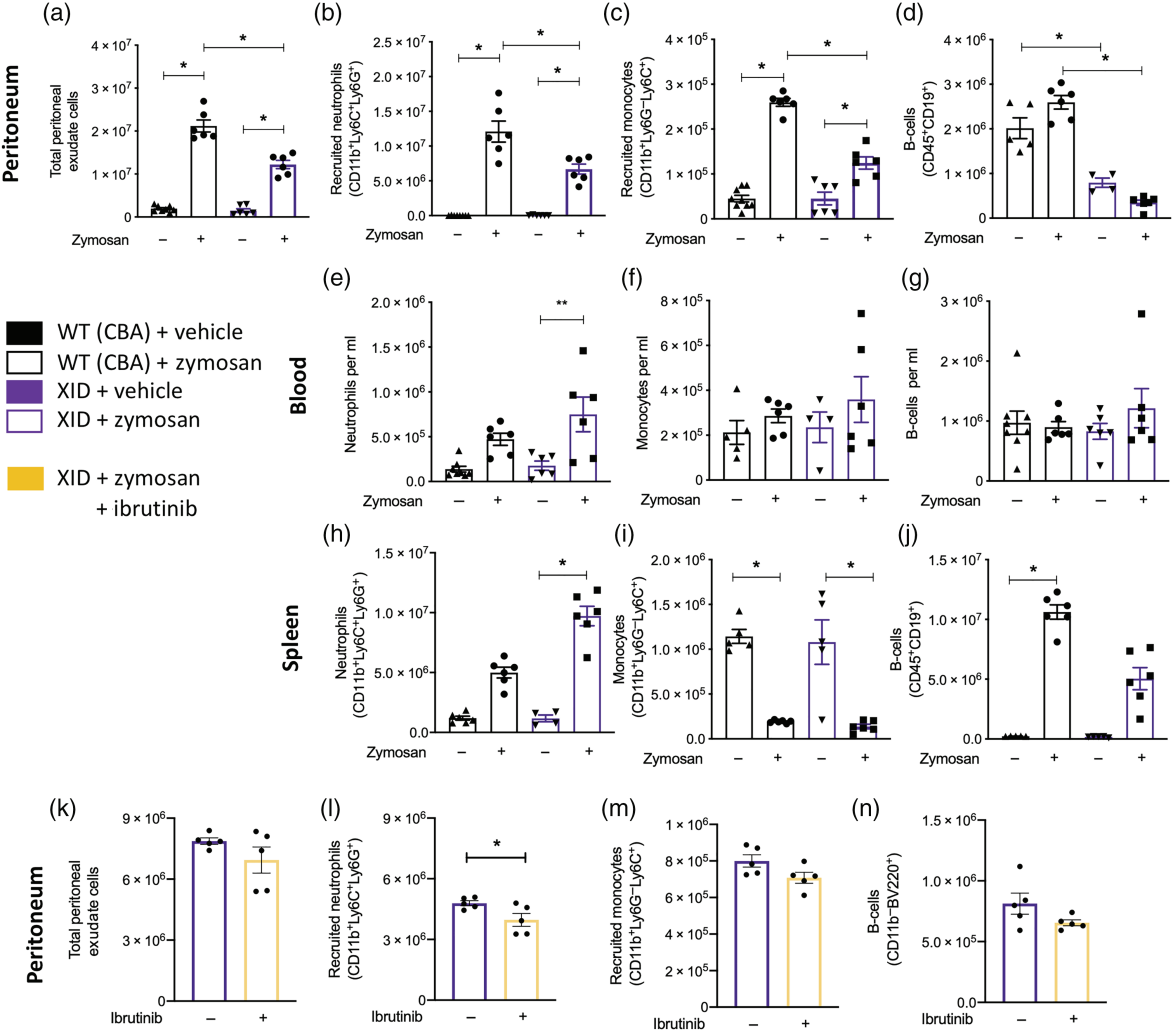
XID mice have reduced myeloid cells recruitment following zymosan challenge. CBA/CaCrl (wild‐type: CBA background) and XID mice (on a CBA background) challenged with zymosan (100 μg; i.p.); after 16‐h peritoneal exudate cells (a–d), blood (e–g) and spleens (h–j) harvested. (a) Total peritoneal exudate cells were quantified, (b) recruited neutrophils (CD11b^+^Ly6C^+^Ly6G^+^), (c) recruited monocytes (CD11b^+^Ly6C^+^Ly6G^−^) and (d) B‐cells (CD11b^−^BV220^+^). Blood was harvested (e) neutrophils (CD11b^+^Ly6C^+^Ly6G^+^) and (f) monocytes (CD11b^+^Ly6C^+^Ly6G^−^) counts assessed and (g) B‐cells (CD11b^−^BV220^+^). (h) Neutrophils (CD11b^+^Ly6C^+^Ly6G^+^), (i) monocytes (CD11b^+^Ly6C^+^Ly6G^−^) and (j) B‐cells (CD11b^−^BV220^+^) counts were assessed. One hour prior to zymosan challenge (100 μg; i.p.), XID mice were pretreated with ibrutinib (10 mg·kg^−1^; p.o.) or vehicle; after 16‐h peritoneal exudate cells were harvested, and (k) total white cells quantified, and (l) neutrophils (CD11b^+^Ly6C^+^Ly6G^+^), (m) monocytes (CD11b^+^Ly6C^+^Ly6G^−^) (n) and B‐cells numbers were assessed. Data shown are means ± SEM of *n* observation mice per group. *P < 0.05; one‐way ANOVA was performed with Bonferroni post hoc test, where there were multiple comparisons or Student's *t*‐test where appropriate

Having shown that multiple BTK inhibitors sequester/prevent monocytes from entering the blood and reduce peritoneal recruitment, we wanted to confirm the effects were BTK specific and not due to a common off‐target effect of the BTK inhibitors used. Therefore, we used XID mice that have a single point mutation in the *Btk* gene rendering the kinase domain inactive. XID mice and WT on the same CBA background were injected with zymosan (100 μg; i.p.), which robustly initiated an acute inflammatory response characterised by an increase in total cellularity within the peritoneum (Figure 4a). Importantly, there was significantly less total cell recruitment to the peritoneum in XID mice injected with zymosan compared with WT mice injected with zymosan at 16 h (Figure 4a). We next characterised the myeloid component of the recruited PECs. When compared with WT mice challenged with zymosan, XID mice challenged with zymosan displayed significantly fewer Ly6C^+^Ly6G^+^ neutrophils (Figure 4b) and Ly6C^+^Ly6G^−^ monocytes (Figure 4c) in the peritoneum at 16 h. XID mice had significantly fewer B‐cells in the peritoneum compared with WT mice, while zymosan challenge did not affect B‐cell numbers (Figure 4d).

Neutrophils and monocytes are released into the circulation from the spleen following an inflammatory stimulus (Swirski et al., 2009); therefore, we wanted to investigate if BTK would regulate neutrophil and monocyte release from the spleen into the blood. We therefore quantified neutrophil and monocyte numbers in the blood and spleen. Following zymosan challenge, there is an increase in the number of neutrophils in the blood in both WT and XID mice 16 h after zymosan challenge (Figure 4e), which is mirrored by an increase in the number of neutrophils in the spleen (Figure 4h). Following zymosan injection, there is no alteration in circulating monocytes number in the blood (Figure 4f); however, there is a significant decrease in the number of monocytes in spleen in both WT and XID mice (Figure 4i). Within the spleen, B‐cell number was significantly increased following zymosan challenge in WT mice but was significantly lower in XID mice; no change in circulating B‐cell levels in blood was observed following zymosan challenge in WT or XID mice (Figure 4g/j). These results suggest that BTK regulates monocyte/neutrophil recruitment to the circulation and to the site of inflammation.

**FIGURE 5:**
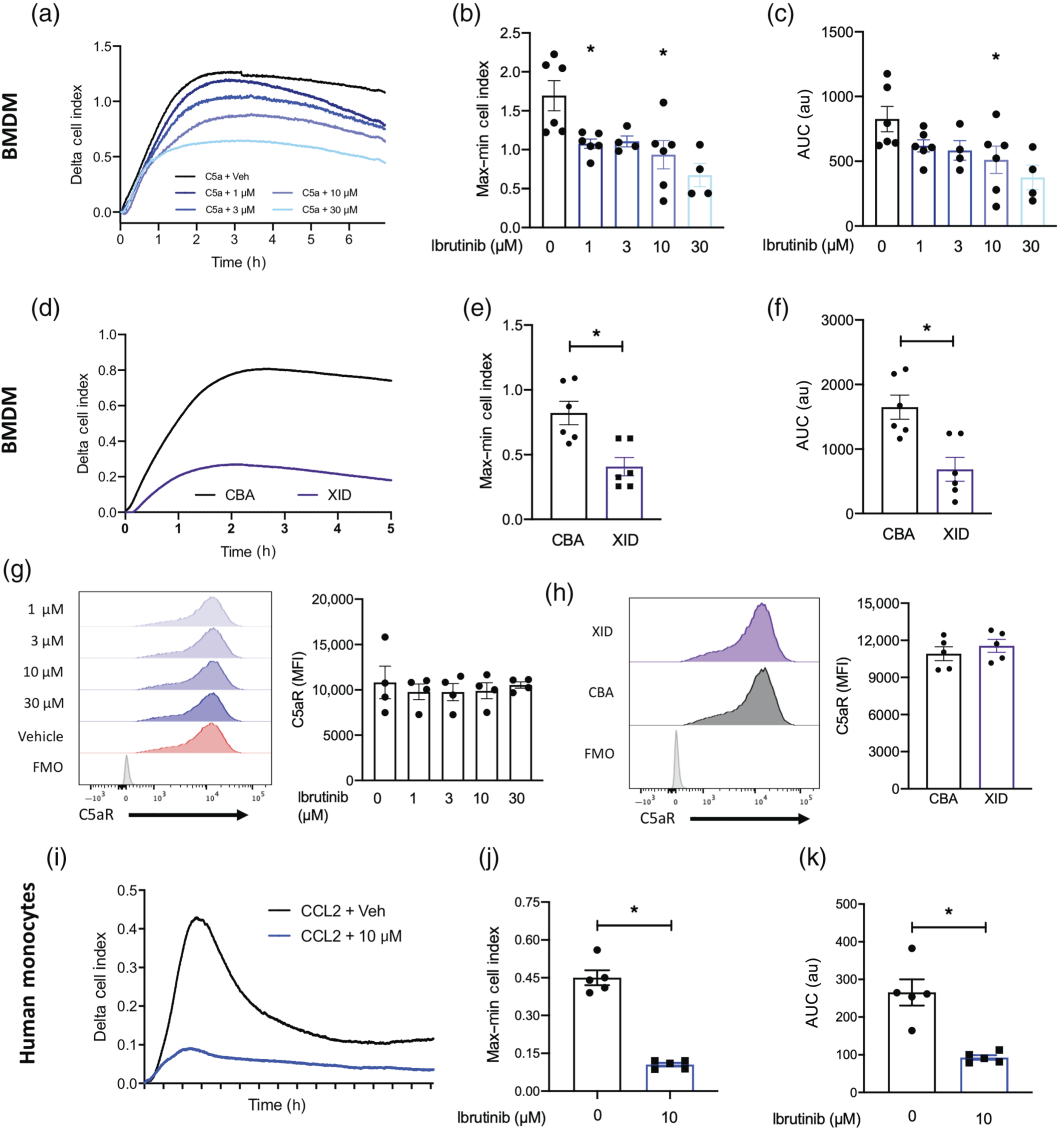
Pharmacological or genetic inhibition of BTK reduces monocyte/macrophage chemotaxis. (a) Bone marrow‐derived macrophages (BMDMs) were incubated with ibrutinib (1–30 μM) for 60 min before being added to the upper chamber (1 × 10^5^ per well) of a CIM‐16 plate and allowed to migrate towards 10 nM of C5a. (b) BMDMs from CBA/CaCrl or XID mice were added to the upper chamber (1 × 10^5^ per well) of a CIM‐16 plate and allowed to migrate towards 10 nM of C5a. (i) Human monocytes were incubated with ibrutinib (10 μM) for 60 min before being added to the upper chamber (4 × 10^5^ per well) of a CIM‐16 plate and allowed to migrate 10 nM of CCL2. Combined traces of *n* = 4–6 biological replicated are shown in panels (a, d, i). Migration was measured with max–min (b, e, j) analysis and AUC (c, f, k). (g) Representative histograph of mean fluorescent intensity (MFI) of C5aR on BMDM treated with ibrutinib or vehicle prior to C5a stimulation and quantified data. (h) Representative histograph of mean fluorescent intensity (MFI) of C5aR on CBA or XID BMDM stimulated with C5a and quantified data. Data are expressed as mean ± SEM, *n* = 5 or 6 biological replicates with 2 technical replicates per condition (a, d, i) or *n* = 5 biological replicated (g and h). Statistical analysis was conducted by one‐way ANOVA with Dunnett's multiple comparison post‐test. *P < 0.05 relative to CCL2 or C5a alone or a Student's *t*‐test where appropriate

We next wanted to confirm that the effects of ibrutinib treatment were BTK specific; to do this, we pretreated XID mice with ibrutinib 1 h prior to zymosan challenge. Importantly, there was no significant difference in cellular recruitment at 16 h in XID mice treated with ibrutinib compared with XID treated with vehicle (Figure 4k); there was a very small but significant decrease in CD11b^+^Ly6C^+^Ly6G^+^ neutrophil number in XID treated with ibrutinib (Figure 4l) and no difference in CD11b^+^Ly6C^+^Ly6G^−^ monocyte recruitment (Figure 5m) and peritoneal B‐cell number (Figure 4n). This finding strongly suggests that BTK signalling is involved in neutrophils and monocyte recruitment during acute inflammation.

### 3.5 BTK regulates monocyte and macrophage chemotaxis

Having shown that signalling through BTK is needed for myeloid cell mobilisation and recruitment in vivo, we wanted to investigate if BTK regulated monocyte/macrophage ability to undergo chemotaxis. To address this question, we used a real‐time chemotaxis assay (Iqbal et al., 2013). Murine BMDMs were pretreated with ibrutinib (1–30 μM) for 60 min and allowed to migrate towards 10 nM complement C5a. Inhibition of BTK with ibrutinib reduced chemotaxis towards C5a in a concentration‐dependent manner (Figure 5a), as quantified by max–min analysis (Figure 5b) and AUC (Figure 5c). To confirm this effect was BTK specific, we generated BMDM from WT or XID mice and compared their ability to undergo chemotaxis towards C5a. BMDM from XID mice had significantly reduced ability to undergo chemotaxis towards C5a (Figure 5d) assessed by max–min analysis (Figure 5e) and AUC (Figure 5f). In vivo we demonstrated that inhibition of BTK did not alter cell surface expression of chemokine receptors CXCR4 on neutrophils and CCR2 in monocytes, we therefore wanted to investigate if inhibiting BTK signalling resulted in reduced expression of C5aR in BMDM treated with ibrutinib. Murine BMDMs were pretreated with ibrutinib (1–30 μM) for 60 min followed by 60 min of stimulation with 10 nM complement C5a. Cell surface expression of C5aR was then assessed by multicolour flow cytometry. Inhibition of BTK using ibrutinib did not alter cell surface expression of C5aR (Figure 5g). Furthermore, there was no significant difference in cell surface expression of C5aR on CBA (WT) and XID BMDM treated with C5a, although statistical tests were not carried out due to small group size (Figure 5h).

Having shown significant effects of BTK on murine macrophage chemotaxis, we next tested the actions of BTK inhibition on human monocyte chemotaxis. Monocytes were isolated by immunomagnetic selection from human leukocyte cones and pretreated with ibrutinib (10 μM) or vehicle 1 h prior to assessment of their migratory capacity. Human monocytes treated with ibrutinib to inhibit BTK displayed significantly reduced ability to undergo chemotaxis towards CCL2 (Figure 5i), assessed by max–min analysis (Figure 5j) and AUC (Figure 5k).

### 3.6 BTK regulates macrophage chemokine production via NF‐κB

**FIGURE 6:**
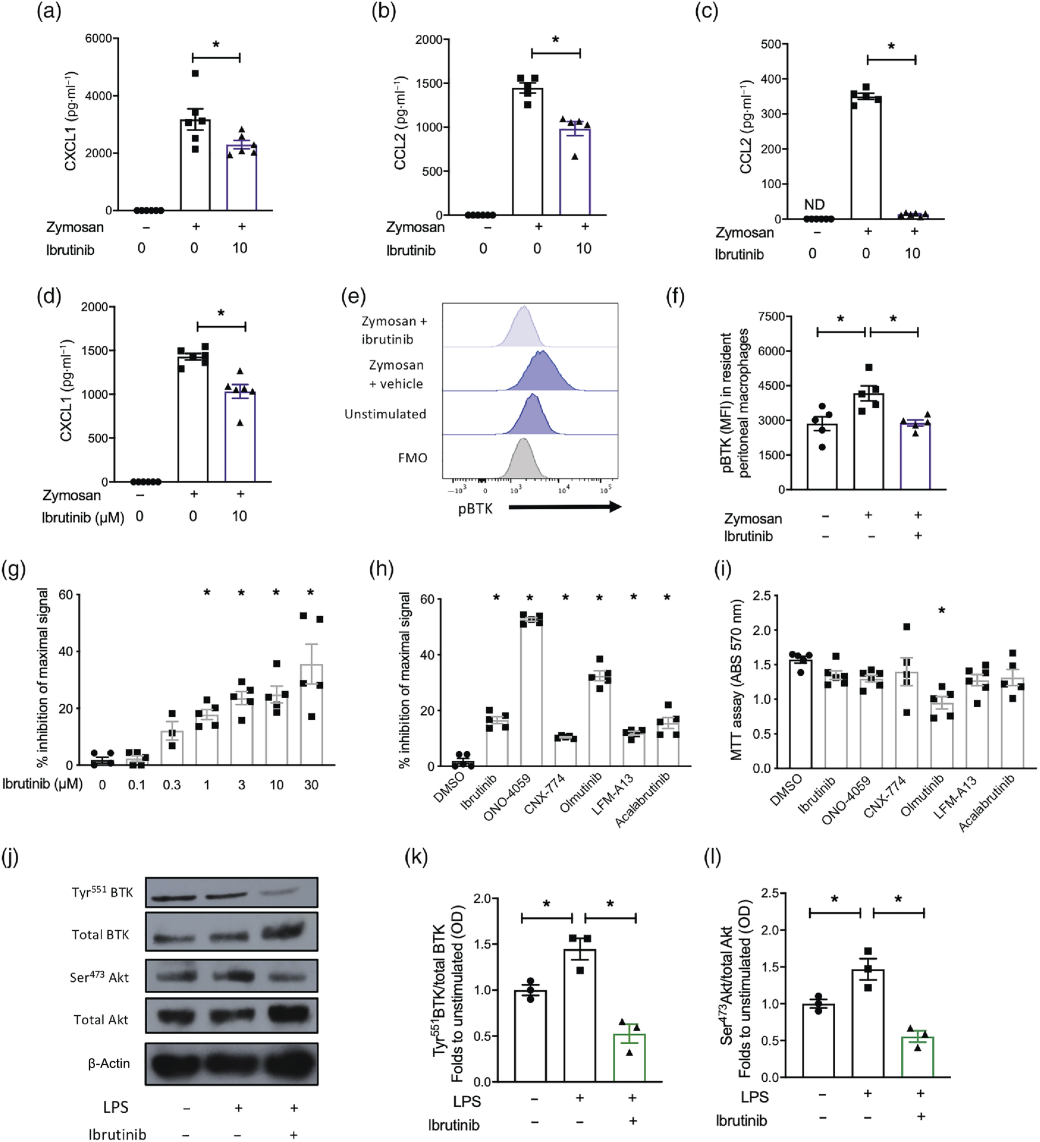
BTK regulates chemokine release from tissue resident macrophages through NF‐κB. (a–c) C57BL/6J mice were pretreated with ibrutinib (10 mg·kg^−1^; p.o.) or vehicle 1 h prior to zymosan challenge (100 μg; i.p.) and peritoneal lavage fluids harvested after 1, 4 and 16 h after zymosan challenge. Levels of chemokines were measured in lavage fluids by ELISA (a) CXCL1 in 1 h, (b) CCL2 at 4 h and (c) CCL2 at 16 h. Data shown are mean ± SEM; *n* = 5 biological repeats. (d) Peritoneal macrophages were isolated from C57BL/6J mice and stimulated ex vivo with zymosan for 1 h and CXCL1 measured by ELISA. (e) Representative histograph of mean fluorescent intensity (MFI) of pBTK on resident peritoneal macrophages pretreated with ibrutinib or vehicle prior to zymosan (10 μg·ml^−1^) stimulation and (f) quantified. (g, h) NF‐κB and AP1 activity assay in RAW Blue cells. Data shown are mean ± SEM; *n* = 5 independent experiments with 4 technical replicates per condition. (g) Concentration response of ibrutinib (0.1–30 μM) pretreatment 1 h prior to LPS stimulation. (h) A range of BTK inhibitors (1 μM) given as a pretreatment 1 h prior to LPS stimulation. (i) MTT assay of RAW Blue cells treated with BTK inhibitors for 6 h at 1 μM. (k, l) BMDMs were pretreated with ibrutinib (1 μM) 1 h prior to LPS stimulation and Tyr^551^ phosphorylation on BTK and Ser^473^ phosphorylation on Akt were assessed by western blot and quantified. Data expressed are mean ± SEM, *n* = 5 or 6 biological replicates (a–f), or *n* = 5 repeat experiments with *n* = 4 technical replicates (g–i) or representative data from *n* = 3 technical repeats (j–l). Statistical analysis was conducted by one‐way ANOVA with Dunnett's multiple comparison post‐test. *P < 0.05 compared with vehicle

We have previously shown that primary macrophages from XID mice or macrophages treated with ibrutinib in vitro secrete less pro‐inflammatory cytokines in response to an inflammatory stimulus (Purvis et al., 2020). Therefore, we wanted to assess if following zymosan challenge in vivo, there was a reduction in chemokine levels in peritoneal lavage fluids. The initial wave of cellular recruitment to the peritoneum is dominated by neutrophils; therefore, we assessed CXCL1 (a potent neutrophil chemoattractant) levels in peritoneal lavage fluids 1 h after zymosan challenge. Mice pretreated with ibrutinib 1 h prior to zymosan challenge had significantly lower CXCL1 levels compared with mice treated with vehicle prior to zymosan challenge (Figure 6a). After the initial neutrophil recruitment, there is an influx of monocytes; therefore, we assessed CCL2 (a potent monocyte chemoattractant) levels in peritoneal lavage fluids after zymosan challenge. Mice pretreated with ibrutinib 1 h prior to zymosan challenge had significantly lower CCL2 levels at 4 (Figure 6b) and 16 h (Figure 6c) when compared with mice treated with vehicle prior to zymosan challenge. To prove that macrophages are the source of CXCL1 levels after zymosan challenge in vivo, we isolated resident peritoneal macrophages and challenged them with zymosan for 1 h then measured secreted chemokine levels. Resident peritoneal macrophages treated with ibrutinib 1 h prior to zymosan challenge had reduced CXCL1 levels compared with vehicle treated (Figure 6d). We next assessed the level of BTK protein phosphorylation in isolated peritoneal macrophages following acute zymosan stimulation ex vivo. Zymosan stimulation resulted in a significant increase in phosphorylation of Tyr^223^ on BTK, which was inhibited with ibrutinib pretreatment (Figure 6e/f).

A key transcription factor that regulates pro‐inflammatory chemokine expression in macrophages is NF‐κB. Therefore, we investigated the role of BTK in regulating NF‐κB activity. Ibrutinib (0.1–30 μM) pretreatment of RAW Blue cells inhibited NF‐κB activity in a concentration‐dependent manner (Figure 6g). We next tested a range of BTK inhibitors (1 μM); all the BTK inhibitors tested significantly inhibited NF‐κB activity (Figure 6h). Only olmutinib showed cellular toxicity at the concentration tested (Figure 6i). We next confirmed the ability of ibrutinib to inhibit phosphorylation of BTK in BMDMs. Pretreatment with ibrutinib 1 h prior to LPS stimulation attenuated the increase in tyrosine phosphorylation on BTK (Figure 6j/k), which also resulted in decreased phosphorylation on Akt (Figure 6k/l). Taken together, these data tell us that inhibition of BTK signalling reduces chemokine secretion from macrophages as a result of reduced NF‐κB activity and Akt signalling.

## 4 DISCUSSION

In this manuscript, we report that inhibition of BTK signalling either pharmacologically or genetically reduces myeloid cell recruitment in acute inflammation. By using multicolour flow cytometry to accurately identify immune cell subsets and by performing a full kinetic analysis, rather than single endpoint approaches, we were able to demonstrate the role of BTK throughout the acute inflammatory response. Our key findings were confirmed using two EMA/FDA approved BTK inhibitors, a range of structurally different BTK inhibitors and BTK‐deficient XID mice. Importantly, we demonstrated that inhibition of BTK using ibrutinib in zymosan challenged mice reduced phosphorylation of BTK on blood Ly6C^hi^ monocytes but not Ly6C^lo^ monocytes, demonstrating cellular specificity, which translated into a reduction in the recruitment of inflammatory Ly6C^hi^ monocytes but not patrolling Ly6C^lo^ monocytes to peritoneum. Finally, we explored the mechanism(s) by which inhibition of BTK reduced myeloid cell recruitment during acute inflammation. We revealed two complementary mechanisms of action: (a) Inhibition of BTK reduced monocyte/macrophage chemotaxis to CCL2 and complement C5a, and (b) inhibition of BTK reduced NF‐κB‐dependent chemokine production from tissue resident macrophages.

Chemokines play a key role in monocyte recruitment and macrophage activation in preclinical models of human diseases characterised by chronic inflammation (McNeill et al., 2017). While genetic knockout mice of individual chemokine receptors provide clear evidence for chemokines playing non‐redundant roles in inflammation, interventional studies in man using chemokine receptor antagonists have proven challenging with multiple drugs that target chemokine receptors failing to progress beyond early phase randomised clinical trials. However, as a caveat to this, Schall and Proudfoot (2011) suggest a lot of the negative data was due to inappropriate target selection and ineffective dosing. As an alternative to targeting single monocyte/macrophage chemoattractant GPCRs for therapeutic benefit, we and others have explored the potential of targeting multiple CC chemokines using the chemokine binding protein (35 K‐Fc) (McNeill et al., 2015) or lipoprotein molecules that inhibit monocyte chemotaxis towards CC chemokine (ApoA1) (Iqbal et al., 2016) or blocking macrophage responses to multiple chemoattractants (netrin) (van Gils et al., 2012). Here, we target the non‐receptor bound intracellular signalling molecule BTK. We clearly demonstrate that both pharmacological (Figures 1, 2, 3) and genetic inhibition of BTK signalling (Figure 4) limit myeloid cell recruitment during acute inflammation.

After an acute inflammatory stimulus, there are usually two waves of immune cell infiltration (Marelli‐Berg & Jangani, 2018). Neutrophils are rapidly recruited with peak infiltration 2–8 h after zymosan challenge; this is then followed by an infiltration of monocytes that peaks 16 h after zymosan challenge. Many anti‐inflammatory drugs have been shown to reduce cellular infiltration in this model (Navarro‐Xavier et al., 2010). Here, we demonstrate that treatment with ibrutinib reduces Tyr^233^ phosphorylation on BTK in blood neutrophils and Ly6C^hi^ monocytes. Importantly, BTK phosphorylation correlated with reduced neutrophils and Ly6C^hi^ monocytes recruitment to the peritoneum. Of note, Ly6C^lo^ monocytes had significantly less phosphorylation of BTK in zymosan + vehicle mice, while treatment with ibrutinib did not significantly reduce Ly6C^lo^ monocyte recruitment to the peritoneum. However, the vast majority of monocytes were recruited in this model are Ly6C^hi^. In this report, we show that BTK inhibitors have potent anti‐inflammatory properties in a widely used model of sterile inflammation. In accordance with our data, de Porto et al. (2019) reported that ibrutinib treatment reduces PMN recruitment in acute pulmonary inflammation evoked by antibiotic‐treated pneumococcal pneumonia and suggested that ibrutinib has the potential to inhibit ongoing lung inflammation in an acute infectious setting. O'Riordan et al. (2020) reported reduced infiltration of neutrophils in a model of polymicrobial sepsis in XID mice. Decreased myeloid cells recruitment has also been reported in other inflammatory models: RA, obesity and cerebral ischaemia, when BTK had been systemically inhibited with BTK inhibitors (Weber et al., 2017). With the exception of CNX‐774, an irreversible BTK inhibitor, all other tested BTK inhibitors reduced cellular recruitment to the peritoneum. CNX‐774 binds BTK covalently with an IC_50_ value of <1 nM. Studies of possible off‐target protein reactivity assays show significant specificity against cellular thiols and plasma proteins (Zhang et al., 2018). Noteworthy, a recent study that addressed BTK inhibition as an approach to block IgE‐mediated histamine release in human basophils found that ibrutinib showed a more potent effect blocking allergen‐induced histamine secretion, when compared with CNX‐774 drug. It has been reported that IgER cross‐linking in basophils is followed by phosphorylation of SYK and that SYK, once activated, is capable of phosphorylating BTK (Smiljkovic et al., 2017). The authors suggest that ibrutinib could be acting by inhibiting other known kinases apart from BTK and these converge to exert their actions in that way. However, this mode of action does not seem entirely plausible in the context of our study as the XID mice have reduced monocyte and neutrophil recruitment in the 16‐h peritonitis model; therefore, caution should be taken in selecting the appropriate BTK inhibitor for future studies. However, taken together, our data suggest strongly that BTK inhibitors may represent novel therapeutic agents that could be used to reduce PMN recruitment in the setting of both acute and chronic inflammation.

One of the first steps in leukocyte recruitment is adhesion to and rolling along the vascular endothelium. While XID mice have reduced neutrophil and monocyte recruitment to the peritoneum, neutrophils also appear not to be recruited as efficiently from the blood. Mueller et al. demonstrated using adhesion under flow experiments that BTK regulated E‐selectin mediated slow rolling of neutrophils. In addition, they reported that downstream signalling of the BTK pathway divided into PLCγ2 and PI3Kγ‐dependent pathways, both of which independently regulated β_2_‐integrin mediated adhesion (Mueller et al., 2010). We have extended these findings further, demonstrating in vivo that genetic and pharmacological inhibition of BTK signalling significantly reduces neutrophil recruitment and monocytes recruitment in vivo, giving further physiological relevance to these findings. It should be noted that BTK most likely is only one of many interdependent mechanisms by which monocytes and neutrophils undergo directed movement along a chemotactic gradient.

Our experiments have shown for the first time that primary murine and human monocytes and macrophages have reduced ability to undergo real‐time chemotaxis to physiological relevant chemoattractants (CCL2 and C5a). Chemotaxis involves the directed movement of cells along a concentration gradient (Rumianek & Greaves, 2020). This movement involves cytoskeletal re‐arrangement directed primarily by small Ras‐like GTPases, cdc42 and Rac1 (Weber et al., 1998). In the formation of lamellipodia, BTK has been shown to co‐localise with Rac1 and Cdc42 (Nore et al., 2000). Additionally, BTK harbours pleckstrin homology domains that allow it to interact with filamentous actin and BTK has been shown to co‐localise with F‐actin (Yao et al., 1999). A note of caution is that the aforementioned work was carried out in B‐cells. However, RNA sequencing data generated from monocytes isolated from healthy donors and patients with X‐linked agammaglobulinemia (XLA) (inactive BTK) demonstrated differentially expressed novel lincRNAs that co‐located with genes related to ‘focal adhesion’ and ‘regulation of actin cytoskeleton’ (Mirsafian et al., 2017). Collectively, these lines of evidence all point towards BTK having a key role in myeloid cell movement, having the ability to reduce cellular recruitment and chemotaxis by around 50%. Our data show that BTK signalling, in part, regulates neutrophil and monocyte recruitment in vivo and their ability to undergo chemotaxis in vitro.

Macrophages are a major source of chemokine production following activation in both acute and chronic inflammation. We have shown that inhibition of macrophage BTK reduces cytokine and chemokine release both in vitro and in vivo in diabetes and poly microbial sepsis (O'Riordan et al., 2020; Purvis et al., 2020). A pro‐inflammatory transcription factor that tightly regulates chemokine production is NF‐κB; XID macrophages have poor induction of NF‐κB following inflammatory stimulation (Mukhopadhyay et al., 2002). In this report, we demonstrate that pharmacological inhibition of BTK reduces NF‐κB and AP1 activity in WT macrophages; NF‐κB is known to be a master transcription factor for the production of pro‐inflammatory chemokine production. Pharmacological inhibition of NF‐κB mediated cytokine and chemokine production has been shown to be beneficial in many acute and chronic preclinical disease models (Chen et al., 2017; Johnson et al., 2017). Another selective BTK inhibitor, CGI1746, has been reported to reduce CCL2 levels from macrophages in myeloid cell‐dependent arthritis by blocking trans‐phosphorylation of BTK Tyr551 and subsequent auto‐phosphorylation at Tyr223 (Di Paolo et al., 2011). Activated BTK trans‐phosphorylates PLCγ2 on Tyr1217, one of the major regulatory residues involved in calcium mobilisation needed for amongst other things cytokine release. Here, we demonstrate that inhibition of BTK may have a beneficial effect in a wide range of inflammatory pathologies in vivo due to reducing chemokine production and therefore reducing myeloid recruitment, which can exacerbate disease progression.

There has been a push in the last number of years to repurpose existing medicines for new therapeutic indications. There are a number of benefits to this strategy as these medications have (a) full safety profiles, (b) significantly reduced cost compared with developing novel medications, (c) reduced cost to health care providers as many medications will be off patent, and (d) clinical data from existing patients who are taking these medications for other indications. The recent COVID‐19 pandemic saw a surge in pre‐existing medications being trialled for the treatment of severe inflammatory syndromes, with the emergence of Dexamethasone (Sterne et al., 2020) and Tocilizumab (RECOVERY Collaborative Group et al., 2021) from the RECOVERY Trial being recommended in the treatment of COVID‐19. Indeed, this opens up the possibility that new BTK inhibitors that are currently in preclinical studies may also be tested in disease modalities other than for B‐cells malignancies (Langrish et al., 2021). Our new data, along with numerous other reports, demonstrate that ibrutinib and acalabrutinib, which are both EMA/FDA approved medications, could be used in a wide range of inflammatory conditions due to their potent anti‐inflammatory effects in myeloid cells, specifically their ability to regulate myeloid cell recruitment and reduce cytokine and chemokine production from macrophages, both of which are very tractable therapeutic targets.

It should be noted that off‐target effects of ibrutinib have been reported to include atrial fibrillation, reoccurring infection and immunosuppression (Weber et al., 2017). Of note, atrial fibrillation is not seen in patients treated with other BTK inhibitors. Atrial fibrillation has been attributed to inhibition of C‐terminal Src kinase (Xiao et al., 2020). Impaired leukocyte/platelet interaction has also been reported (Nicolson, Nock, et al., 2020); however, this could be advantageous following acute myocardial infraction. Longer term use of TK inhibitors is known to result in resistance. However, activation of myeloid cells is a key process in the pathology of many acute and chronic diseases so limiting this could have numerous advantages, but the most likely new use of a BTK inhibitor will in the treatment of acute inflammatory conditions, for example, sepsis, abdominal aortic aneurysm (AAA) or myocardial infraction.

Patients who develop acute lymphoblastic leukaemia (ALL) take BTK inhibitors (Wen et al., 2021). One implication of the data presented in this paper is that inhibiting BTK in monocytes and neutrophils, as an off‐target drug effect, could result in their inefficient recruitment of innate immune cells to sites of ongoing infection and result in reduced pathogen clearance. Of note, patients who are on ibrutinib report increased incidence of any Grade 3–5 infection, while patients with XLA also have increased incidence of bacterial and fungal infection (Ball et al., 2020). Our data are also complementary to findings that demonstrate BTK inhibitors have promise in preclinical work in models of rheumatoid arthritis. Monocyte/macrophage infiltration into the synovial fluid has been correlated with disease severity (Gómez‐Aristizábal et al., 2019); one could envisage that, if a BTK inhibitor was used, there would be reduced monocyte and neutrophil infiltration, therefore reduced inflammation in the joint; however, more work is needed to confirm this hypothesis.

In conclusion, we have demonstrated a novel role for BTK in regulating myeloid cell recruitment during acute inflammation. Specifically, we demonstrate a non‐redundant role of BTK signalling in neutrophil and monocyte recruitment in a self‐resolving model of sterile inflammation. Inhibition of BTK was able to modulate myeloid cell recruitment by two independent but complementary mechanisms: (a) reducing monocyte chemotaxis to CCL2 and (b) reducing chemokine production by tissue resident macrophages. Our data strengthen the case for using BTK inhibitors to reduce monocyte infiltration and macrophage activation in acute inflammatory diseases like sepsis, rheumatoid arthritis or cardiovascular disease including myocardial infarction or stroke.

## AUTHOR CONTRIBUTIONS

GSDP and DRG conceptualised the study. GSDP and HAT did the experimental work and analysed the data. GSDP drafted the manuscript. GSDP, HAT, KC and DRG reviewed and edited the manuscript.

## CONFLICT OF INTEREST

The authors declare no conflict of interest.

## DECLARATION OF TRANSPARENCY AND SCIENTIFIC RIGOUR

This Declaration acknowledges that this paper adheres to the principles for transparent reporting and scientific rigour of preclinical research as stated in the *BJP* guidelines for Design and Analysis, Immunoblotting and Immunochemistry, and Animal Experimentation, and as recommended by funding agencies, publishers and other organisations engaged with supporting research.

## Supporting information




**Figure S1:** Full gating strategies for myeloid and B‐cells analysis.

**Figure S2:** Effect of ibrutinib treatment on peritoneal B‐cell number, and recruited Ly6C^hi^ and Ly6C^lo^ numbers.
C57BL/6J mice were pre‐treated with increasing dose of ibrutinib (0.01–30 mg/kg; p.o.) one hour prior to zymosan challenge (100 μg; i.p.) and peritoneal exudate cells harvested after 16 h. **(A)** B‐cell number in the peritoneal exudate. (B) number of Ly6C^hi^ monocytes (CD11b^+^Ly6G^−^CD115^+^Ly6C^hi^) and (C) number of Ly6C^lo^ monocytes (CD11b^+^Ly6G^−^CD115^+^Ly6C^lo^). Data shown are means ± SEM of *n* = 5 mice per group. *P < 0.05; one‐way ANOVA was performed with Bonferroni post hoc test, where there were multiple comparisons.

## Data Availability

The data that support the findings of this study are available from the corresponding author upon reasonable request. Some data may not be made available because of privacy or ethical restrictions.
