# Supplementary figures and images for: Bruton's TK regulates myeloid cell recruitment during acute inflammation
Source: Br J Pharmacol. 2022 Mar 15;179(11):2754–70. doi: 10.1111/bph.15778 (PMC9361009; doi:10.1111/bph.15778)

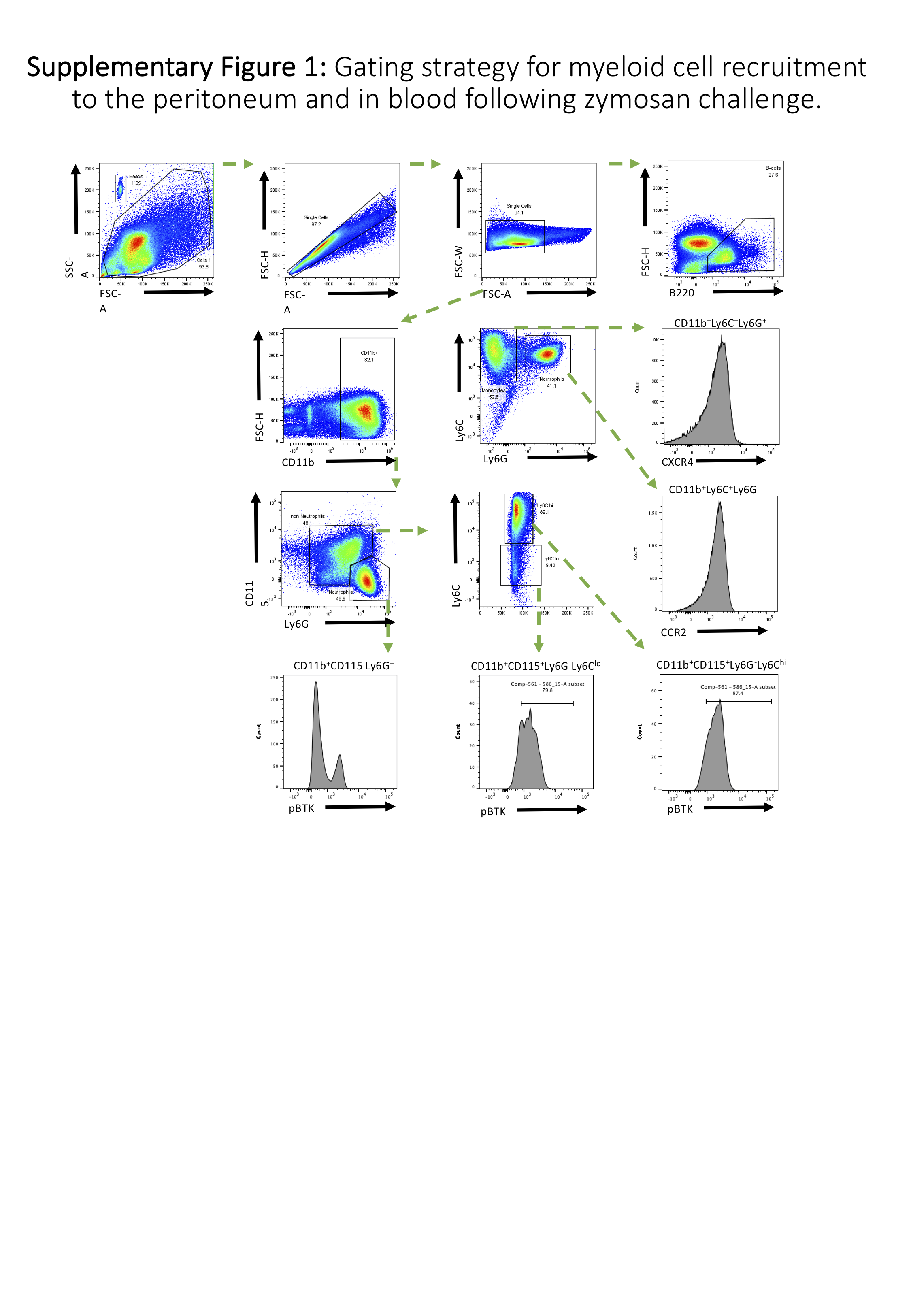

Supplement: Supplementary file 1 — Figure S1: Full gating strategies for myeloid and B‐cells analysis. [file BPH-179-2754-s001.tiff]

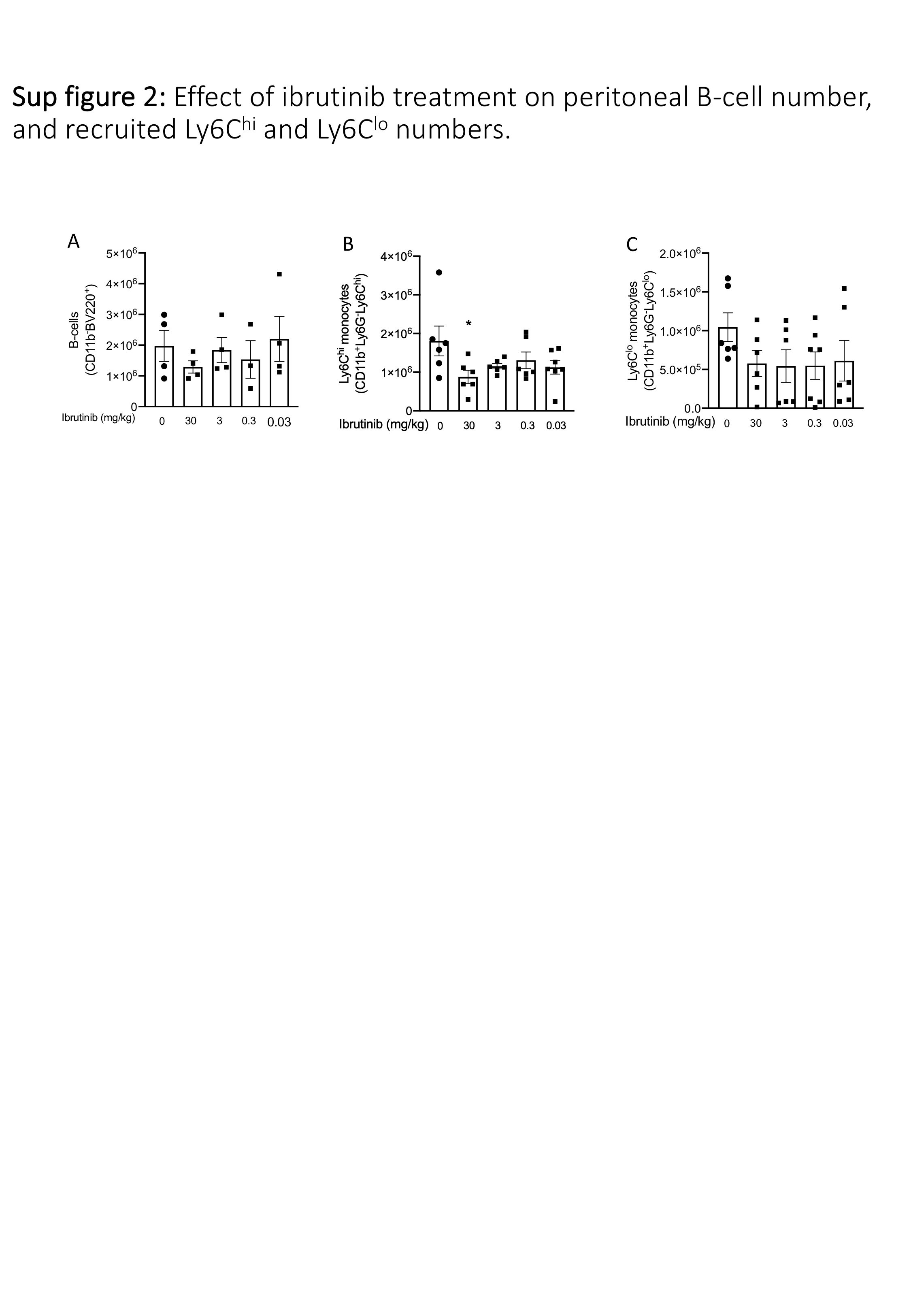

Supplement: Supplementary file 2 — Figure S2: Effect of ibrutinib treatment on peritoneal B‐cell number, and recruited Ly6Chi and Ly6Clo numbers. C57BL/6J mice were pre‐treated with increasing dose of ibrutinib (0.01–30 mg/kg; p.o.) one hour prior to zymosan challenge (100 μg; i.p.) and peritoneal exudate cells harvested after 16 h. (A) B‐cell number in the peritoneal exudate. (B) number of Ly6Chi monocytes (CD11b+Ly6G−CD115+Ly6Chi) and (C) number of Ly6Clo monocytes (CD11b+Ly6G−CD115+Ly6Clo). Data shown are means ± SEM of n = 5 mice per group. *P < 0.05; one‐way ANOVA was performed with Bonferroni post hoc test, where there were multiple comparisons. [file BPH-179-2754-s002.tiff]
